# Submaximal cardiopulmonary thresholds on a robotics-assisted tilt table, a cycle and a treadmill: a comparative analysis

**DOI:** 10.1186/s12938-015-0099-0

**Published:** 2015-11-10

**Authors:** Jittima Saengsuwan, Tobias Nef, Marco Laubacher, Kenneth J. Hunt

**Affiliations:** Division of Mechanical Engineering, Department of Engineering and Information Technology, Institute for Rehabilitation and Performance Technology, Bern University of Applied Sciences, Pestalozzistrasse 20, 3400 Burgdorf, Switzerland; ARTORG Center for Biomedical Engineering Research, Gerontechnology and Rehabilitation Research Group, University of Bern, Bern, Switzerland; Department of Physical Medicine and Rehabilitation, Faculty of Medicine, Khon Kaen University, Khon Kaen, Thailand

**Keywords:** Robotics-assisted tilt table, Treadmill, Cycle ergometer, Submaximal exercise, Anaerobic threshold, Respiratory compensation point, Cardiopulmonary exercise testing

## Abstract

**Background:**

The robotics-assisted tilt table (RATT), including actuators for tilting and cyclical leg movement, is used for rehabilitation of severely disabled neurological patients. Following further engineering development of the system, i.e. the addition of force sensors and visual bio-feedback, patients can actively participate in exercise testing and training on the device. Peak cardiopulmonary performance parameters were previously investigated, but it also important to compare submaximal parameters with standard devices. The aim of this study was to evaluate the feasibility of the RATT for estimation of submaximal exercise thresholds by comparison with a cycle ergometer and a treadmill.

**Methods:**

17 healthy subjects randomly performed six maximal individualized incremental exercise tests, with two tests on each of the three exercise modalities. The ventilatory anaerobic threshold (VAT) and respiratory compensation point (RCP) were determined from breath-by-breath data.

**Results:**

VAT and RCP on the RATT were lower than the cycle ergometer and the treadmill: oxygen uptake (V′O_2_) at VAT was [mean (SD)] 1.2 (0.3), 1.5 (0.4) and 1.6 (0.5) L/min, respectively (p < 0.001); V′O_2_ at RCP was 1.7 (0.4), 2.3 (0.8) and 2.6 (0.9) L/min, respectively (p = 0.001). High correlations for VAT and RCP were found between the RATT vs the cycle ergometer and RATT vs the treadmill (R on the range 0.69–0.80). VAT and RCP demonstrated excellent test–retest reliability for all three devices (ICC from 0.81 to 0.98). Mean differences between the test and retest values on each device were close to zero. The ventilatory equivalent for O_2_ at VAT for the RATT and cycle ergometer were similar and both were higher than the treadmill. The ventilatory equivalent for CO_2_ at RCP was similar for all devices. Ventilatory equivalent parameters demonstrated fair-to-excellent reliability and repeatability.

**Conclusions:**

It is feasible to use the RATT for estimation of submaximal exercise thresholds: VAT and RCP on the RATT were lower than the cycle ergometer and the treadmill, but there were high correlations between the RATT vs the cycle ergometer and vs the treadmill. Repeatability and test–retest reliability of all submaximal threshold parameters from the RATT were comparable to those of standard devices.

## Background

A robotics-assisted tilt table (RATT) provides safe mobilization and intensive sensorimotor stimulation for early rehabilitation of neurological patients by tilting the patient upright and implementing cyclical leg stepping movement. The RATT has separate actuators for tilting the table and for continuously moving the legs during therapy.

The RATT device employed in the present work is a clinical product (Erigo, Hocoma AG, Switzerland), which as standard includes neither measurement of the patient’s work rate, nor does it provide the patient with any form of biofeedback which could be used to guide their active participation. To extend the functionality of the standard RATT, specifically to make it possible to implement formal exercise testing protocols on the device, the RATT was augmented with force sensors and a visual bio-feedback system [1]. The force sensors were inserted under the leg cuffs which attach the patient’s legs to the leg-drive systems. Using additional measurements of the moment arms and the joint angular velocities, the true work rate (in Watts) applied by the patient at the human–machine interface can be calculated in real time. The new visual biofeedback system which was added to the standard device shows the patient a target work rate and, in real time, the actual, measured work rate. The patient is instructed to adapt their volition leg effort, by producing forces into the leg cuffs in synchrony with the cyclical leg motion, in order to follow the work rate target as closely as possible. The target work rate can be chosen arbitrarily, but, for exercise testing purposes, it will be a standardized test protocol such as a constant work rate or incremental ramp. These engineering extensions have enabled severely disabled neurological patients to actively participate in exercise testing and training on the RATT [2, 3].

The augmented RATT device, with the engineering developments outlined above, makes possible for the first time the implementation of standardized exercise testing protocols on a robotics-assisted tilt table for determination of the key parameters of cardiopulmonary status (testing) and to allow optimized prescription of exercise regimes (training). An incremental exercise test, where the patient’s work rate increases linearly over a short time period, delivers two types of parameters: (1) peak cardiopulmonary performance parameters (peak oxygen uptake and peak heart rate), which characterize aerobic capacity, and (2) submaximal exercise thresholds (primarily the ventilatory anaerobic threshold, VAT, and respiratory compensation point, RCP), which serve mainly to allow prescription of training intensity.

We previously reported on peak cardiopulmonary performance parameters (parameter group (1), above) obtained using the augmented RATT, and compared peak responses from the augmented RATT with standard modalities (treadmill and cycle ergometers) [4]. In the present work, we investigate the second major parameter group, (2) above, which can be obtained from incremental exercise testing, viz. the submaximal exercise thresholds VAT and RCP, together with several secondary submaximal parameters. The submaximal parameters from the RATT are directly compared with values obtained in the same subjects using treadmill and cycle ergometers. This investigation is considered clinically relevant because most neurological patients such as those with stroke or multiple sclerosis often terminate exercise testing before their maximal effort is reached. Non-cardiopulmonary factors, such as cognitive problems, muscle weakness or fatigue, are the causes linked to exercise termination in these patients [5, 6].

The submaximal exercise thresholds, i.e. the oxygen uptake at the ventilatory anaerobic threshold (V′O_2@VAT_) and at the respiratory compensation point (V′O_2@RCP_), are important because they can provide crucial information for the assessment of fitness status [7–9] or for exercise prescription [10–12]. They are independent of subjects’ motivation [13] and the duration of the exercise testing protocol [14]. Furthermore, V′O_2@VAT_ is reported to be useful for follow up after an intervention [15–17], for the prediction of all-cause postoperative mortality [18] and for the assessment of the severity of heart failure [19].

Other submaximal exercise parameters derived from ventilation (V′E), such as ventilatory equivalent of oxygen (V′E/V′O_2_), ventilatory equivalent of carbon dioxide (V′E/V′CO_2_) and the V′E-vs-V′CO_2_ slope, provide additional information regarding the existence and severity of heart and lung diseases [20, 21]. Additionally, V′E/V′CO_2_ and the V′E-vs-V′CO_2_ slope are important predictors for mortality in some groups of patients, e.g. patients with heart failure [22, 23].

Numerous studies reported differences in submaximal exercise parameters on the cycle ergometer and the treadmill [14, 24–26], the arm ergometer and the cycle ergometer [27, 28], and the arm ergometer, the cycle ergometer and the treadmill [29]. It has been shown that the submaximal thresholds, e.g. V′O_2@VAT_, from the arm ergometer were lower than the cycle ergometer, and V′O_2@VAT_ from the cycle ergometer was lower than the treadmill [14, 24, 27, 28]. Regarding submaximal exercise parameters such as V′E/V′CO_2_ and the V′E-vs-V′CO_2_ slope, there are conflicting data. Sun et al. reported no mode-dependent difference in V′E/V′CO_2_ and the V′E-vs-V′CO_2_ slope [26]; however, Davis et al. found that V′E/V′CO_2_ and the V′E-vs-V′CO_2_ slope were higher on the treadmill than the cycle ergometer in women but not in men, and concluded that women demonstrated mode dependency in ventilatory efficiency indices [25].

Since there are no previous data regarding the comparative evaluation of submaximal exercise parameters from the RATT, the aim of this study was to evaluate the feasibility of the RATT for estimation of submaximal exercise thresholds and to compare these with the cycle ergometer and the treadmill.

## Methods

### Study design and selection criteria

This descriptive study was reviewed and approved by the Ethics Review Committee of the Swiss Canton of Bern, Switzerland (Reference No. 002/12). All research subjects gave their written informed consent before participating in the study.

Subjects were included in the study if they were 18–50 years and had no history of cardiovascular, pulmonary and musculoskeletal disease that might have interfered with the exercise testing.

### Testing procedures

Subjects were randomly assigned to perform six maximal individualized incremental exercise tests, with two tests on each of the three exercise modalities: a treadmill (Venus, h/p/cosmos GmbH, Germany—2 tests), a cycle ergometer (LC7, Monark Exercise AB, Sweden—2 tests) and a robotics-assisted tilt table (RATT; Erigo, Hocoma AG, Switzerland—2 tests) [4]. Each test session was separated by at least 48 h but not more than 7 days and the time of day was controlled. Subjects were advised to avoid strenuous activity for at least 24 h and not to consume food for at least 3 h before the exercise testing [30].

**Fig. 1:**
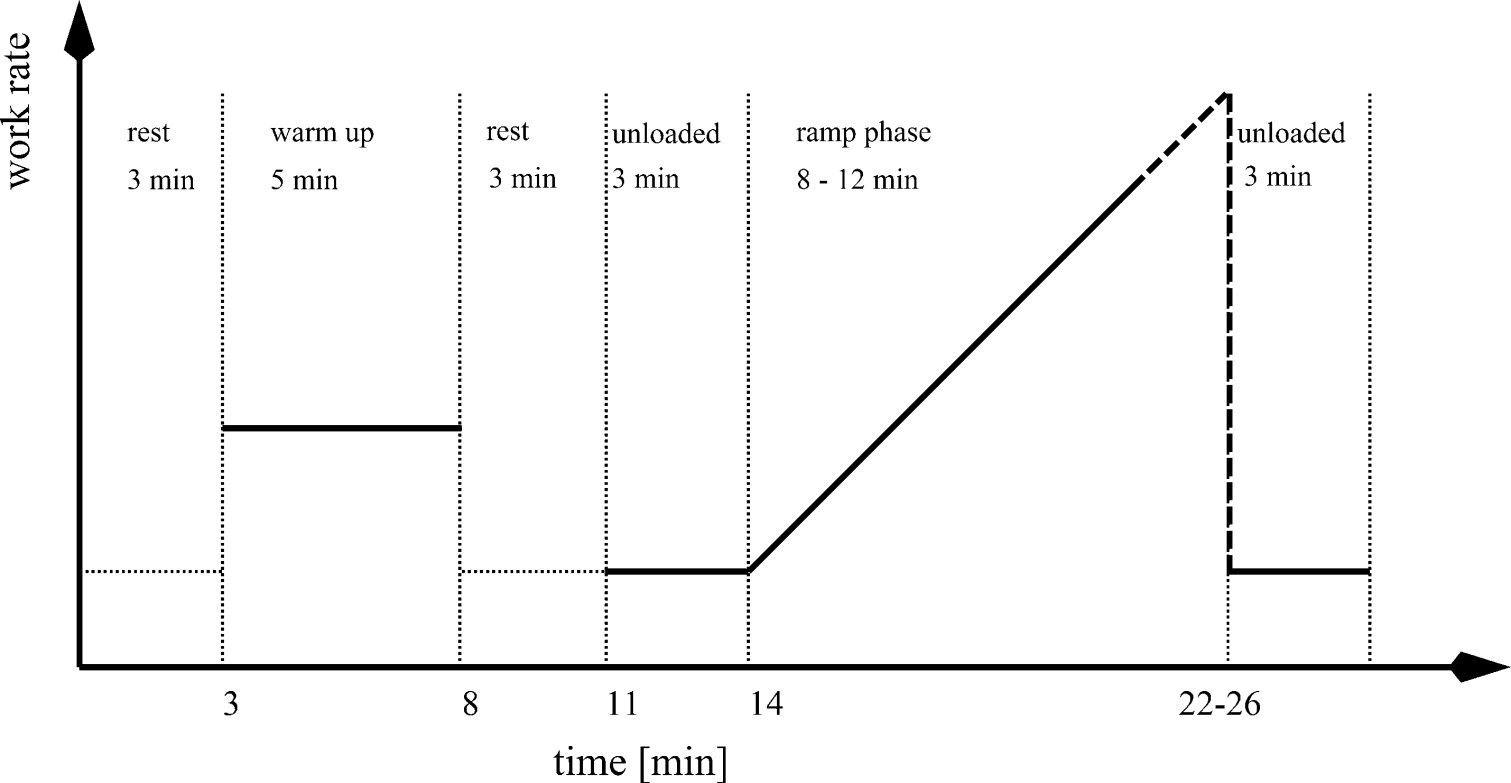
Incremental exercise testing protocol for all three devices

The incremental exercise testing protocol on each device was the same: it started with 3 min of rest, 5 min of warm up, 3 min of rest, and 3 min of unloaded movement before the ramp phase (Fig. 1). Subjects’ work rate increments during the ramp phase were estimated from their predicted maximal oxygen uptake [31] in order that the subjects would reach their maximal exercise performance in 8–12 min [14].

**Fig. 2:**
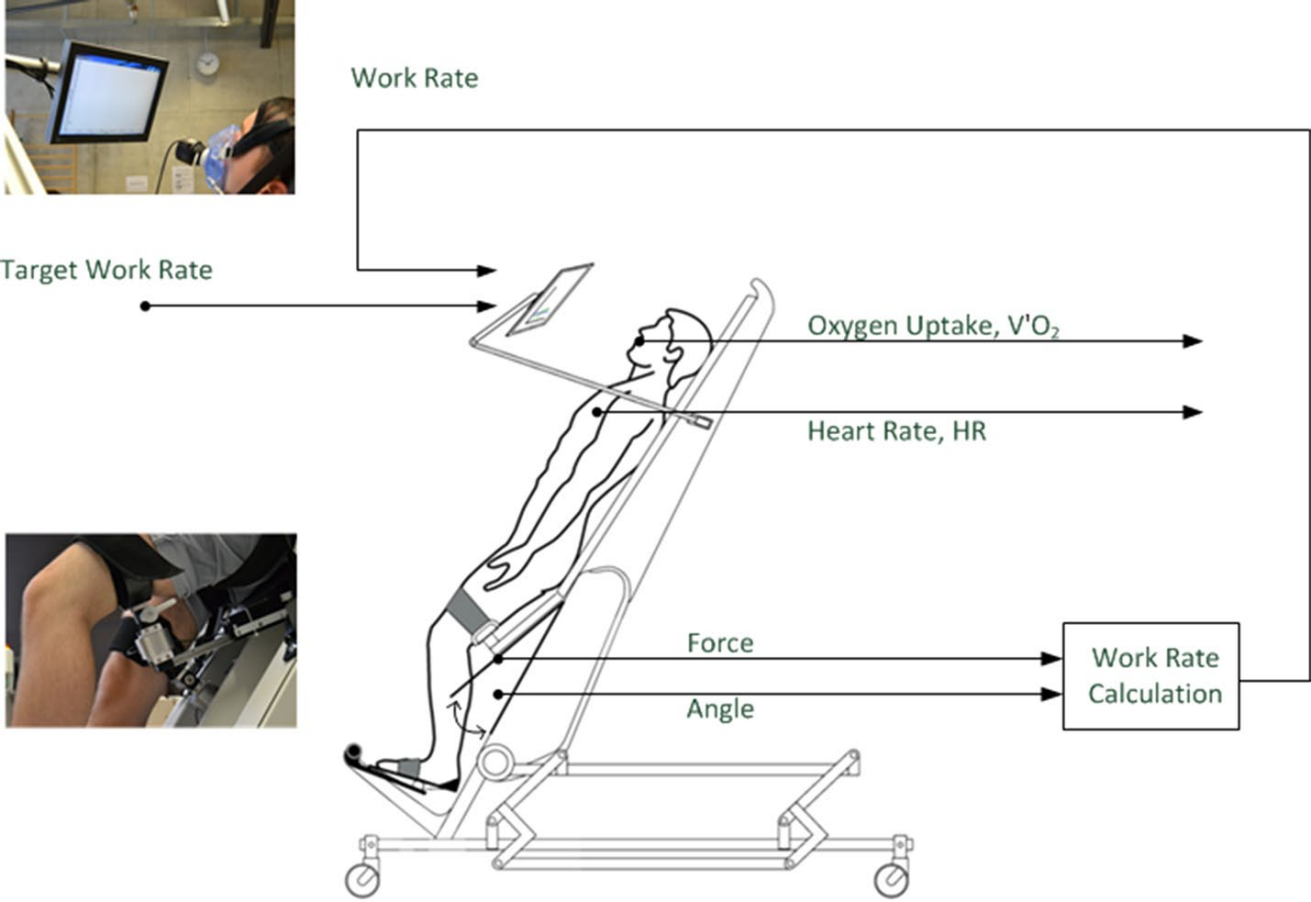
Robotics-assisted tilt table (RATT) with visual feedback system. The visual feedback screen shows the target work rate and the subject’s work rate. The subject’s work rate was calculated from the forces in the thigh cuffs and the angular velocities

RATT: The subjects were first secured with a body harness, thigh cuffs and foot straps. Then the RATT was tilted to 70 degrees and the stepping movement was set at 80 steps/min, which is the maximal achievable step rate on this device. The RATT ramp rate was set in the range of 4 to 12 W/min. The subjects were instructed to actively push into the leg cuffs to produce force to follow the target work rate which they could see and compare to their actual work rate in real time (Fig. 2).

Cycle ergometer: The ramp rate ranged from 12 to 40 W/min. The settings for the seating and pedalling were adjusted for each subject and recorded to ensure the same position in subsequent tests.

Treadmill: During the ramp phase, the work rate increment ranged from 14 to 30 W/min. The work rate was increased linearly every 30 s using combined non-linear changes in speed and slope [32].

Cardiopulmonary data were recorded with a breath-by-breath cardiopulmonary testing system (MetaMax 3B, Cortex Biophysik GmbH, Germany). Before each test, full calibration was performed: pressure calibration; volume calibration with a 3-L syringe; and two-point gas calibration using ambient air and a precision gas mixture (15 % oxygen and 5 % carbon dioxide). Heart rate was recorded using a chest strap (model T34, Polar Electro Oy, Finland). The cardiopulmonary variables were analysed using a 15-breath average on the corresponding Metasoft software (version 2.7.29, Cortex Biophysik GmbH, Germany) [33].

### Outcome measures

The VAT and RCP were identified according to the criteria suggested by Binder et al. [11]. The VAT was visually determined using the combination of these approaches: (1) the point of deflection of V′CO_2_ versus V′O_2_ (V-slope method) [34]; (2) the point where V′E/V′O_2_ reaches its minimum or starts to rise without a rise in V′E/V′CO_2_; and, (3) the point at which partial pressure of end-tidal oxygen tension (P_ET_O_2_) reaches a minimum or starts to rise without a decline in the partial pressure of end-tidal carbon dioxide tension (P_ET_CO_2_).

The RCP was visually determined by: (1) the point of deflection of V′E versus V′CO_2_; (2) the minimal value or nonlinear rise of V′E/V′CO_2_; and, (3) the point that P_ET_CO_2_ starts to decline.

The above approaches were used to determine the values of V′O_2_ and V′E/V′O_2_ at VAT (V′O_2@VAT_ and V′E/V′O_2@VAT_), and V′O_2_ and V′E/V′CO_2_ at RCP (V′O_2@RCP_ and V′E/V′CO_2@RCP_). The slope of V′E-vs-V′CO_2_ from the start of the ramp phase to the RCP was also estimated.

### Statistical analysis

Normality of the data was assessed by the Shapiro–Wilk test. Data from the second tests on each device were used for the comparative and correlation analyses. Repeated measures analysis of variance (ANOVA) was conducted to determine whether there were differences of V′O_2@VAT_ and V′O_2@RCP_ between the three devices. If a statistically significant difference was found, Bonferroni post hoc multiple comparison corrections were applied to examine differences between each paired data set.

Linear regression analysis was used to identify the correlation between the values of V′O_2@VAT_ and V′O_2@RCP_ on the RATT vs cycle ergometer and vs treadmill. The regression equation, correlation coefficient (R), coefficient of determination (R^2^) and standard error of the estimate (SEE) were obtained.

Test–retest reliability of submaximal parameters on each device was analysed using a 2-way mixed single measures (absolute agreement) intraclass correlation coefficient (ICC_3,1_) [35]. 0.40 ≤ ICC < 0.75 was considered as fair to good reliability and ICC ≥0.75 was considered excellent reliability [36]. Repeatability was analysed using the Bland and Altman limits of agreement, incorporating mean difference and coefficient of repeatability [37]. The within-subject coefficients of variation were also calculated [38]. The test–retest reliability was based on only nine subjects because of a technical problem in the measurement device detected in the data from the first tests in eight subjects.

All analyses were performed using SPSS (Version 19.0, IBM Corp.).

## Results

Seventeen subjects were included (9 male, 8 female). The subjects had the following characteristics [mean (SD))]: age 28.4 (6.4) years, height 171.8 (9.8) cm, body mass 68.1 (12.5) kg and body mass index 22.6 (2.2) kg/m^2^.

### VAT and RCP

The VAT was able to be identified in all subjects on all three devices. The RCP on the RATT was identified in 10 subjects (58.8 %), on the cycle ergometer in 17 subjects (100 %), and on the treadmill in 15 subjects (88.2 %); in 9 subjects, the RCP was identified for all three devices.

**Fig. 3:**
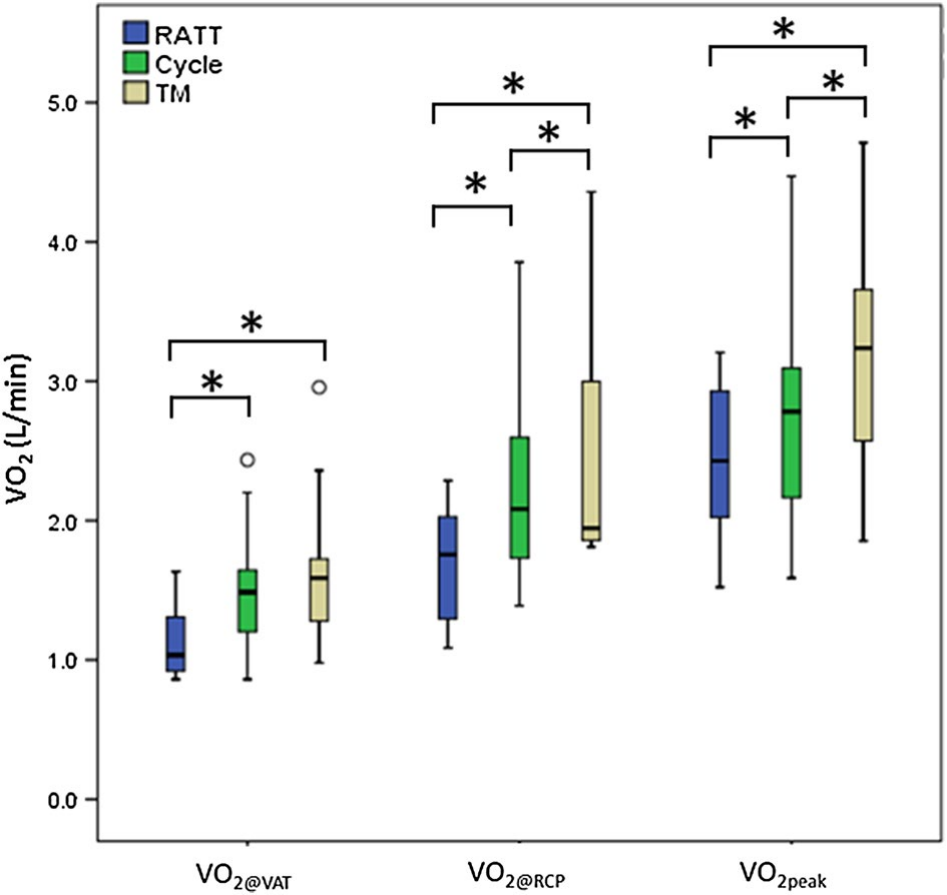
Box plots for VO_2@VAT_, VO_2@RCP_ and VO_2peak_ among the 3 devices.* Asterisks* represent significant differences in each paired data set assessed by Bonferroni post hoc multiple comparison corrections

The V′O_2@VAT_ and V′O_2@RCP_ from the RATT were lower than the cycle ergometer and the treadmill: absolute V′O_2@VAT_ from the RATT, the cycle ergometer and the treadmill was [mean (SD)] 1.2 (0.3), 1.5 (0.4) and 1.6 (0.5) L/min, respectively (p < 0.001); V′O_2@RCP_ from the RATT, the cycle ergometer and the treadmill was 1.7 (0.4), 2.3 (0.8) and 2.6 (0.9) L/min, respectively (p = 0.001) (Table 1; Fig. 3). On average, the V′O_2@VAT_ on the RATT was 21.4 % lower than the cycle ergometer V′O_2@VAT_ and 26.1 % lower than the treadmill V′O_2@VAT_ (mean individual differences). The V′O_2@RCP_ on the RATT was 23.9 % lower than the cycle ergometer V′O_2@RCP_ and 30.6 % lower than the treadmill V′O_2@RCP_ (mean individual differences).

**Table 1:** Submaximal performance parameters from the RATT, cycle and treadmill (VAT: n = 17; RCP: n = 9)

Variables	RATT	Cycle ergometer	Treadmill	*P* value
V′O_2peak_ absolute (L/min)^a,b,c^ (n = 17)	2.39 ± 0.6	2.82 ± 0.8	3.2 ± 0.9	<0.001
Absolute V′O_2@VAT_ (L/min)^a,b^ (n = 17)	1.16 ± 0.3	1.53 ± 0.4	1.64 ± 0.5	<0.001
Relative V′O_2@VAT_ (mL/kg/min)^a,b^	17.2 ± 3.6	22.3 ± 4.0	23.8 ± 4.7	<0.001
V′O_2@VAT_ as % of V′O_2peak_	49.4 ± 8.8	54.5 ± 5.1	50.6 ± 5.9	0.047
HR at VAT (beats/min)^a^	114.3 ± 12.9	125.3 ± 10.6	121.7 ± 12.8	0.007
HR at VAT as percent predicted HR_peak_ (%)^a^	59.6 ± 6.3	65.4 ± 4.8	63.5 ± 5.9	0.007
Absolute V′O_2@RCP_ (L/min)^a,b,c^ (n = 9)	1.68 ± 0.4	2.26 ± 0.8	2.55 ± 0.9	0.001
Relative V′O_2@RCP_ (mL/kg/min)^a,b^	24.9 ± 5.1	33.0 ± 7.5	37.3 ± 9.3	<0.001
V′O_2@RCP_ as % of V′O_2peak_^a^	68.7 ± 10.2	78.5 ± 9.9	79.1 ± 15.6	0.022
HR at RCP (beats/min)^a^	141.3 ± 17.5	153.2 ± 18.1	158.7 ± 21.9	0.004
HR at RCP as percent predicted HR_peak_ (%)^a^	73.6 ± 7.1	79.8 ± 6.9	82.6 ± 9.0	0.003
V′E/V′O_2@VAT_^b,c^ (n = 17)	23.6 ± 2.9	23.8 ± 3.0	21.8 ± 2.2	0.002
V′E/V′CO_2@RCP_ (n = 9)	28.9 ± 2.3	27.5 ± 2.8	26.7 ± 2.9	0.022
V′E-vs-V′CO_2_ slope to RCP^a,b^ (n = 9)	28.4 ± 2.8	26.4 ± 2.4	25.7 ± 2.7	0.002

Data are given as mean ± standard deviation

*V′O*_*2peak*_ peak oxygen uptake, *V′O*_*2*_ oxygen uptake, *VAT* ventilatory anaerobic threshold, *V′O*_*2@VAT*_ V′O_2_ at VAT, *HR* heart rate, *HR*_*peak*_ peak heart rate, *RCP* respiratory compensation point, *V′O*_*2@RCP*_ V′O_2_ at RCP, *V′E/V′O*_*2@VAT*_ ventilatory equivalent of oxygen at VAT, *V′E/V′CO*_*2@RCP*_ ventilatory equivalent of carbon dioxide at RCP, *V′E-vs-V′CO*_*2*_ *slope* ventilation versus carbon dioxide output slope

^a^ *p* < 0.05 between the RATT and the cycle ergometer

^b^ *p* < 0.05 between the RATT and the treadmill

^c^ *p* < 0.05 between the cycle ergometer and the treadmill

**Fig. 4:**
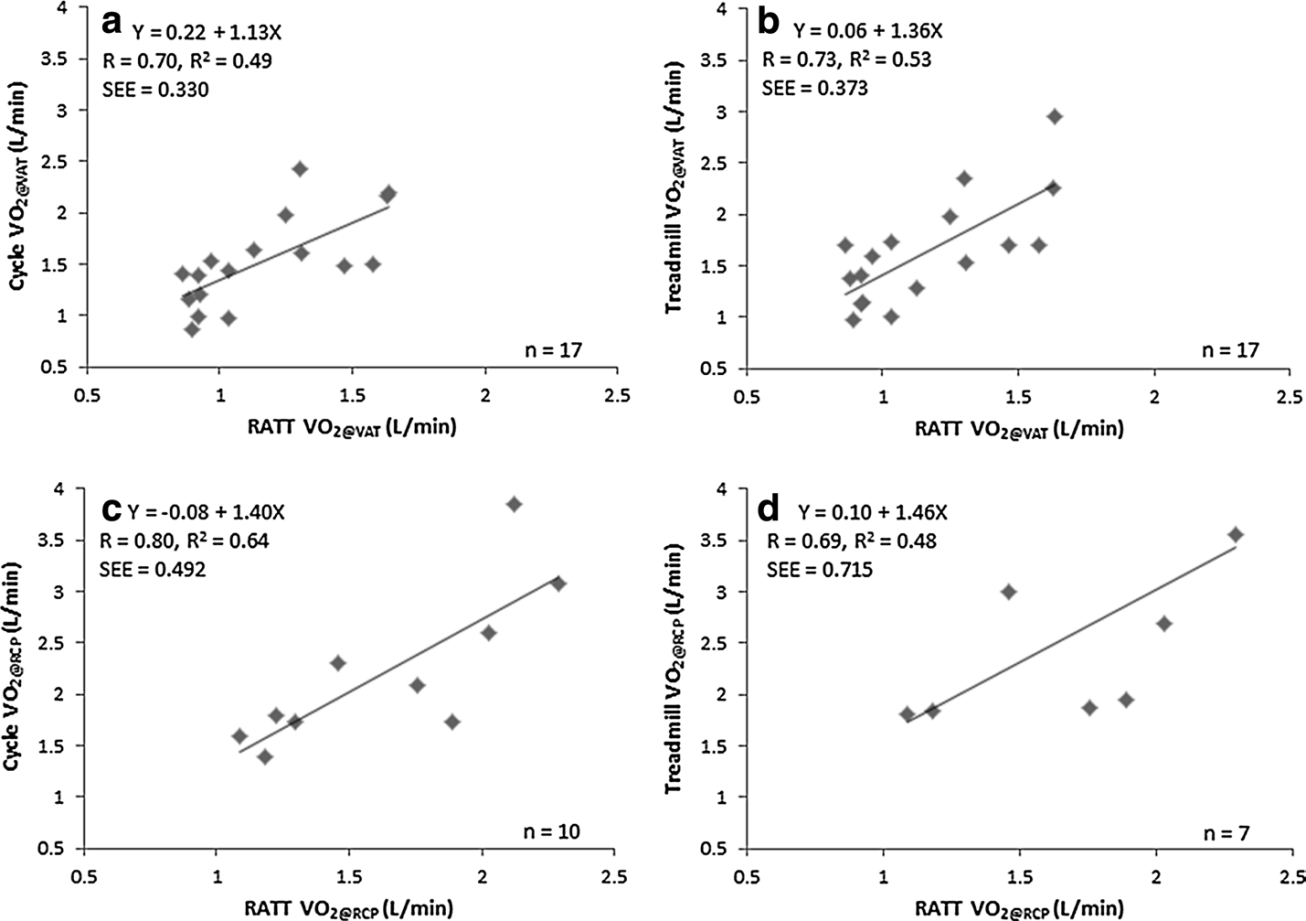
Linear regression analysis of V′O_2@VAT_ (**a**, **b**) and V′O_2@RCP_ (**c**, **d**) on the RATT vs the cycle ergometer and the RATT vs the treadmill

High correlations were found between the RATT vs the cycle ergometer V′O_2@VAT_ (R = 0.70, p < 0.01) and V′O_2@RCP_ (R = 0.80, p < 0.01). The RATT vs the treadmill V′O_2@VAT_ (R = 0.73, p < 0.01) and V′O_2@RCP_ (R = 0.69, p < 0.05) demonstrated similarly high correlations (Fig. 4). The V′O_2@VAT_ and V′O_2@RCP_ demonstrated excellent test–retest reliability for all three devices (ICC 0.81–0.98). The mean differences between the test and retest values on each device were close to zero (Table 2).

**Table 2:** Test-retest reliability of the submaximal performance parameters from the RATT, cycle and treadmill

	Overall mean (tests 1 and 2)	MD (95 % LoA)	CoV (%)	ICC (95 % CI)	SEM	%SEM
V′O_2@VAT_ (L/min) (n = 9)						
RATT	1.129	−0.047 (−0.225, 0.131)	5.91	0.92 (0.60–0.98)	0.064	5.67
Cycle ergometer	1.411	0.082 (−0.459, 0.624)	14.10	0.81 (0.39–0.95)	0.195	13.82
Treadmill	1.561	−0.015 (−0.290, 0.259)	6.78	0.98 (0.90–1.0)	0.099	6.34
V′O_2@RCP_ (L/min)						
RATT (n = 6)	1.649	0.094 (−0.208, 0.395)	6.52	0.92 (0.52–0.99)	0.109	6.61
Cycle ergometer (n = 8)	2.344	−0.014 (−0.706, 0.678)	8.66	0.87 (0.47–0.97)	0.245	10.45
Treadmill (n = 7)	2.609	−0.069 (−0.640, 0.502)	7.17	0.95 (0.74–0.99)	0.206	7.90
V′E/V′O_2@VAT_ (n = 9)						
RATT	23.7	−0.01 (−3.5, 3.5)	5.02	0.70 (0.10–0.93)	1.26	5.31
Cycle ergometer	23.4	1.46 (−4.3, 7.2)	9.78	0.62 (0.05–0.90)	2.07	8.87
Treadmill	21.7	0.20 (−2.5, 2.9)	4.33	0.74 (0.19–0.94)	0.97	4.47
VE/V′CO_2@RCP_						
RATT (n = 6)	28.8	0.70 (−1.0, 2.4)	2.48	0.77 (0.03–0.97)	0.62	2.16
Cycle ergometer (n = 8)	27.7	1.24 (−1.2, 3.6)	4.80	0.90 (0.33–0.98)	0.86	3.10
Treadmill (n = 7)	26.4	−0.97 (−4.6, 2.6)	5.23	0.71 (0.09–0.94)	1.30	4.92
V′E-vs-V′CO_2_ slope to RCP						
RATT (n = 6)	28.0	0.73 (−4.0, 5.5)	6.00	0.53 (−0.38–0.92)	1.71	6.11
Cycle ergometer (n = 8)	26.4	2.19 (−2.0, 6.4)	8.26	0.75 (0.04–0.95)	1.52	5.77
Treadmill (n = 7)	25.4	−1.14 (−4.3, 2.1)	5.13	0.74 (0.12–0.95)	1.15	4.54

*MD* mean difference, *LoA* limits of agreement, *CoV* coefficient of variation, *ICC* intraclass correlation coefficient, *CI* confidence interval, *SEM* standard error of the measurement

### Other parameters

V′E/V′O_2@VAT_ from the RATT and the cycle ergometer were comparable and both were higher than the treadmill. There were no significant differences in V′E/V′CO_2@RCP_. The V′E-vs-V′CO_2_ slope to RCP on the RATT was higher than the cycle ergometer and the treadmill (Table 1). V′E/V′O_2@VAT_, V′E/V′CO_2@RCP_ and V′E-vs-V′CO_2_ slope to RCP had coefficients of variation less than 10 %, the ICC ranged from 0.53 to 0.92 and the repeatability of the parameters, demonstrated by the mean difference, on the RATT and the treadmill were lower than the cycle ergometer (Table 2).

## Discussion

The aim of this study was to evaluate the feasibility of the RATT for estimation of submaximal exercise thresholds by comparison with a cycle ergometer and a treadmill.

### Submaximal exercise thresholds

We found that V′O_2@VAT_ and V′O_2@RCP_ were lower on the RATT than on the cycle and the treadmill. These findings are in line with the differences in V′O_2peak_ found between three devices: the V′O_2peak_ from the RATT was approximately 20 % lower than the cycle V′O_2peak_ and 30 % lower than the treadmill V′O_2peak_ [4]. The lower V′O_2_ may be explained by the lower muscle mass needed to exercise on the RATT. Additionally, the subjects may be less familiar with the stepping movement on the RATT compared with the movement on the standard devices [4].

High correlations were found between V′O_2@VAT_ and V′O_2@RCP_ on the RATT vs the treadmill and on the RATT vs the cycle ergometer; however, the correlation coefficients found were lower than those for the V′O_2peak_ between devices (R = 0.94–0.95) [4]. The difference in the level of correlations found may be related to differences in muscle groups and muscle fibre types used during submaximal exercise on each device for each individual [7]. The correlations we found for V′O_2@VAT_ are higher than in studies of arm ergometer vs cycle ergometer in normal subjects (0.60–0.64) [27, 28], which may reflect the closer pattern of movement on the RATT vs the cycle ergometer compared with different muscles used for exercise on the arm ergometer.

We found no pairwise differences in V′O_2@VAT_ as a percentage of V′O_2peak_ among the three devices. The V′O_2@VAT_ (%V′O_2peak_) found here, 49–55 %, is consistent with some studies on the treadmill or the cycle ergometer (range from 47 to 58 %) [14, 24, 27, 39–41], and consistent with the observation that V′O_2@VAT_ (%V′O_2peak_) rarely exceeds 60 % of V′O_2peak_ [12]. However, other studies reported higher V′O_2@VAT_ (%V′O_2peak_) (range from 60.4 to 77 %) in normal subjects [19, 42, 43]. The difference between V′O_2@VAT_ (%V′O_2peak_) among studies may be caused by the difference in methods of identifying V′O_2@VAT_, the gender, the fitness level and the age distribution of the subjects studied. It was found that V′O_2@VAT_ occurs at a higher percentage of V′O_2peak_ in older subjects, in women and in well-trained subjects [12, 20, 44, 45].

The V′O_2@RCP_ as a percentage of V′O_2peak_ on the RATT was approximately 10 % lower than on the treadmill. In general, data regarding V′O_2@RCP_ (%V′O_2peak_) are less well established compared to V′O_2@VAT_ (%V′O_2peak_). The V′O_2@RCP_ (%V′O_2peak_) identified is in accordance with previous reports [42, 46, 47]. The lower proportion of subjects whose RCP could be identified on the RATT than the cycle or the treadmill may reflect that the RATT is less consistent in provoking cardiorespiratory loads high enough to reach RCP.

We found excellent test–retest reliability in submaximal exercise thresholds (ICC 0.81–0.98). The test–retest reliability of submaximal exercise thresholds obtained from the RATT was comparable to the treadmill and the cycle ergometer. The test–retest reliability for submaximal exercise thresholds found here were slightly lower than for peak oxygen uptake (ICC 0.97–0.99) [4]. The lower test–retest reliability in submaximal exercise thresholds than in peak oxygen uptake has been demonstrated both in normal subjects, and cardiac and pulmonary patients [48–50]. One possible explanation is that the submaximal exercise thresholds may be more sensitive to day-to-day biological variability [51, 52].

### Other parameters

Although there was a trend toward higher V′E/V′CO_2@RCP_ on the RATT, the pairwise comparison did not reach statistical significance. V′E/V′O_2@VAT_ and V′E-vs-V′CO_2_ slope to RCP on the RATT were higher than on the cycle and the treadmill. A study on the arm ergometer found significant differences in V′E/V′O_2@VAT_ (27.7 and 22.1) and V′E/V′CO_2@VAT_ (29.7 and 25.7) between the arm ergometer and the cycle ergometer, respectively [27]. The lower ventilatory efficiency of the RATT and arm ergometer confirms that mode dependency in ventilatory efficiency indices exists. Therefore, the device used for exercise testing should be considered in the analysis of the ventilatory efficiency data. Apart from the arm ergometer, there are no data regarding the ventilatory efficiency in alternative exercise devices for a comparison of results. Most studies on alternative devices focused more on the peak cardiopulmonary values [53, 54] or submaximal values of V′O_2_ or heart rate [55–57]. Since ventilatory efficiency data could provide additional information regarding the severity and prognosis of some heart or lung diseases [20–23], more study of these parameters on the alternative exercise testing devices should be done.

The test–retest reliability of the ventilatory efficiency was fair to excellent. The coefficient of variation was less than 10 %. This is consistent with Davis et al. who found that the test–retest reliability of the V′E/V′CO_2_ and V′E-vs-V′CO_2_ slope to RCP were high [58].

Our study has some limitations. Firstly, the RCP on the RATT could be identified in only 10/17 subjects. This may be because of the limitation that the RATT can elicit lower cardiopulmonary responses compared to the cycle ergometer and the treadmill in healthy subjects. Since this device is mainly intended to be used in patients with severe disability, this may not be a problem in the target population. Secondly, it cannot be verified whether the differences in submaximal exercise thresholds on each device would be the same for severely disabled neurological patients because it is not possible to implement the exercise tests on standard devices (e.g. treadmill) in severely disabled patients. Finally, the sample size was small, but the results provide preliminary estimates to support further study in target patient populations.

## Conclusion

The results suggest that it is feasible to use the RATT for estimation of submaximal exercise thresholds: although V′O_2@VAT_ and V′O_2@RCP_ from the RATT were lower than the cycle ergometer and the treadmill, there were high correlations demonstrated between the RATT vs the cycle ergometer and vs the treadmill; furthermore, the repeatability and test–retest reliability of all submaximal threshold parameters from the RATT were comparable to those of standard devices. There was evidence of mode-dependent differences in V′E/V′O_2@VAT_ and V′E-vs-V′CO_2_ slope to RCP.

## Abbreviations

CI: confidence interval
CoV: coefficient of variation
HR: heart rate
HR_peak_: peak heart rate
ICC: intraclass correlation coefficient
LoA: limits of agreement
MD: mean difference
P_ET_CO_2_: partial pressure of end-tidal carbon dioxide tension
P_ET_O_2_: partial pressure of end-tidal oxygen tension
R: correlation coefficient
R^2^: coefficient of determination
RATT: robotics-assisted tilt table
RCP: respiratory compensation point
SEE: standard error of the estimate
SEM: standard error of measurement
VAT: ventilatory anaerobic threshold
V′CO_2_: carbon dioxide output
V′E: minute ventilation
V′E/V′CO_2_: ventilatory equivalent of carbon dioxide
V′E/V′O_2_: ventilatory equivalent of oxygen
V′O_2_: oxygen uptake
V′O_2peak_: peak oxygen uptake

## References

[1] Bichsel L, Sommer M, Hunt KJ (2011). Development of a biofeedback system for controlling the patients work rate, heart rate and oxygen uptake during robot-assisted tilt table therapy. Automatisierungstechnik.

[2] Laubacher M, Perret C, Hunt KJ (2015). Work-rate-guided exercise testing in patients with incomplete spinal cord injury using a robotics-assisted tilt-table. Disabil Rehabil Assist Technol.

[3] Saengsuwan J, Huber C, Schreiber J, Schuster-Amft C, Nef T, Hunt KJ (2015). Feasibility of cardiopulmonary exercise testing and training using a robotics-assisted tilt table in dependent-ambulatory stroke patients. J Neuroeng Rehabil..

[4] Saengsuwan J, Nef T, Laubacher M, Hunt KJ (2015). Comparison of peak cardiopulmonary performance parameters on a robotics-assisted tilt table, a cycle and a treadmill. PLoS One.

[5] Tang A, Eng JJ, Tsang TS, Krassioukov AV (2013). Cognition and motor impairment correlates with exercise test performance after stroke. Med Sci Sports Exerc.

[6] Koseoglu BF, Gokkaya NK, Ergun U, Inan L, Yesiltepe E (2006). Cardiopulmonary and metabolic functions, aerobic capacity, fatigue and quality of life in patients with multiple sclerosis. Acta Neurol Scand.

[7] American Thoracic Society; American College of Chest Physicians (2003). ATS/ACCP Statement on cardiopulmonary exercise testing. Am J Respir Crit Care Med.

[8] Maciejczyk M, Szymura J, Cempla J, Gradek J, Wiecek M, Bawelski M (2014). Respiratory compensation point during incremental test in overweight and normoweight boys: is it useful in assessing aerobic performance? A longitudinal study. Clin Physiol Funct Imaging.

[9] Weston SB, Gabbett TJ (2001). Reproducibility of ventilation of thresholds in trained cyclists during ramp cycle exercise. J Sci Med Sport..

[10] Mezzani A, Hamm LF, Jones AM, McBride PE, Moholdt T, Stone JA (2013). Aerobic exercise intensity assessment and prescription in cardiac rehabilitation: a joint position statement of the European Association for Cardiovascular Prevention and Rehabilitation, the American Association of Cardiovascular and Pulmonary Rehabilitation and the Canadian Association of Cardiac Rehabilitation. Eur J Prev Cardiol.

[11] Binder RK, Wonisch M, Corra U, Cohen-Solal A, Vanhees L, Saner H (2008). Methodological approach to the first and second lactate threshold in incremental cardiopulmonary exercise testing. Eur J Cardiovasc Prev Rehabil.

[12] Meyer T, Lucia A, Earnest CP, Kindermann W (2005). A conceptual framework for performance diagnosis and training prescription from submaximal gas exchange parameters–theory and application. Int J Sports Med.

[13] Agostoni P, Bianchi M, Moraschi A, Palermo P, Cattadori G, La Gioia R (2005). Work-rate affects cardiopulmonary exercise test results in heart failure. Eur J Heart Fail.

[14] Buchfuhrer MJ, Hansen JE, Robinson TE, Sue DY, Wasserman K, Whipp BJ (1983). Optimizing the exercise protocol for cardiopulmonary assessment. J Appl Physiol Respir Environ Exerc Physiol.

[15] Sullivan MJ, Higginbotham MB, Cobb FR (1989). Exercise training in patients with chronic heart failure delays ventilatory anaerobic threshold and improves submaximal exercise performance. Circulation.

[16] Nishijima H, Sato I, Matsumura N, Mikami T, Nishida M, Yonezawa K (1993). Ventilatory anaerobic threshold before and after cardiac valve surgery. Jpn Circ J.

[17] Ready AE, Quinney HA (1982). Alterations in anaerobic threshold as the result of endurance training and detraining. Med Sci Sports Exerc.

[18] Wilson RJ, Davies S, Yates D, Redman J, Stone M (2010). Impaired functional capacity is associated with all-cause mortality after major elective intra-abdominal surgery. Br J Anaesth.

[19] Matsumura N, Nishijima H, Kojima S, Hashimoto F, Minami M, Yasuda H (1983). Determination of anaerobic threshold for assessment of functional state in patients with chronic heart failure. Circulation.

[20] Wasserman K, Hansen JE, Sue DY, Casaburi R, Whipp BJ (1999). Principles of exercise testing and interpretation.

[21] Sun XG, Hansen JE, Oudiz RJ, Wasserman K (2001). Exercise pathophysiology in patients with primary pulmonary hypertension. Circulation.

[22] Arena R, Myers J, Aslam SS, Varughese EB, Peberdy MA (2004). Peak VO2 and VE/VCO2 slope in patients with heart failure: a prognostic comparison. Am Heart J.

[23] Myers J, Arena R, Oliveira RB, Bensimhon D, Hsu L, Chase P (2009). The Lowest VE/VCO2 ratio during exercise as a predictor of outcomes in patients with heart failure. J Card Fail..

[24] Porszasz J, Casaburi R, Somfay A, Woodhouse LJ, Whipp BJ (2003). A treadmill ramp protocol using simultaneous changes in speed and grade. Med Sci Sports Exerc.

[25] Davis JA, Tyminski TA, Soriano AC, Dorado S, Costello KB, Sorrentino KM (2006). Exercise test mode dependency for ventilatory efficiency in women but not men. Clin Physiol Funct Imaging.

[26] Sun XG, Hansen JE, Garatachea N, Storer TW, Wasserman K (2002). Ventilatory efficiency during exercise in healthy subjects. Am J Respir Crit Care Med.

[27] Orr JL, Williamson P, Anderson W, Ross R, McCafferty S, Fettes P (2013). Cardiopulmonary exercise testing: arm crank vs cycle ergometry. Anaesthesia.

[28] Loughney L, West M, Pintus S, Lythgoe D, Clark E, Jack S (2014). Comparison of oxygen uptake during arm or leg cardiopulmonary exercise testing in vascular surgery patients and control subjects. Br J Anaesth.

[29] Davis JA, Vodak P, Wilmore JH, Vodak J, Kurtz P (1976). Anaerobic threshold and maximal aerobic power for three modes of exercise. J Appl Physiol.

[30] Pina IL, Balady GJ, Hanson P, Labovitz AJ, Madonna DW, Myers J (1995). Guidelines for clinical exercise testing laboratories. A statement for healthcare professionals from the Committee on Exercise and Cardiac Rehabilitation, American Heart Association. Circulation.

[31] Jurca R, Jackson AS, LaMonte MJ, Morrow JR, Blair SN, Wareham NJ (2005). Assessing cardiorespiratory fitness without performing exercise testing. Am J Prev Med.

[32] Hunt KJ (2008). Treadmill control protocols for arbitrary work rate profiles combining simultaneous nonlinear changes in speed and angle. Biomed Signal Process Control.

[33] Robergs RA, Dwyer D, Astorino T (2010). Recommendations for improved data processing from expired gas analysis indirect calorimetry. Sports Med.

[34] Beaver WL, Wasserman K, Whipp BJ (1986). A new method for detecting anaerobic threshold by gas exchange. J Appl Physiol.

[35] Weir JP (2005). Quantifying test-retest reliability using the intraclass correlation coefficient and the SEM. J Strength Cond Res..

[36] Rosner B (2010). Fundamentals of biostatistics.

[37] Bland JM, Altman DG (1986). Statistical methods for assessing agreement between two methods of clinical measurement. Lancet.

[38] Bland JM, Altman DG (1996). Measurement error proportional to the mean. BMJ.

[39] Lucia A, Rivero JL, Pérez M, Serrano AL, Calbet JA, Santalla A (2002). Determinants of VO(2) kinetics at high power outputs during a ramp exercise protocol. Med Sci Sports Exerc.

[40] Habedank D, Reindl I, Vietzke G, Bauer U, Sperfeld A, Gläser S (1998). Ventilatory efficiency and exercise tolerance in 101 healthy volunteers. Eur J Appl Physiol Occup Physiol.

[41] Nordrehaug JE, Danielsen R, Stangeland L, Rosland GA, Vik-Mo H (1991). Respiratory gas exchange during treadmill exercise testing: reproducibility and comparison of different exercise protocols. Scand J Clin Lab Invest.

[42] Loe H, Steinshamn S, Wisløff U (2014). Cardio-respiratory reference data in 4631 healthy men and women 20–90 years: the HUNT 3 Fitness Study. PLoS One.

[43] Herdy AH, Uhlendorf D (2011). Reference values for cardiopulmonary exercise testing for sedentary and active men and women. Arq Bras Cardiol.

[44] Itoh H, Ajisaka R, Koike A, Makita S, Omiya K, Kato Y (2013). Heart rate and blood pressure response to ramp exercise and exercise capacity in relation to age, gender, and mode of exercise in a healthy population. J Cardiol.

[45] Green JM, Crews TR, Bosak AM, Peveler WW (2003). A comparison of respiratory compensation thresholds of anaerobic competitors, aerobic competitors and untrained subjects. Eur J Appl Physiol.

[46] Oussaidene K, Prieur F, Tagougui S, Abaidia A, Matran R, Mucci P (2015). Aerobic fitness influences cerebral oxygenation response to maximal exercise in healthy subjects. Respir Physiol Neurobiol.

[47] Fontana FY, Keir DA, Bellotti C, De Roia GF, Murias JM, Pogliaghi S (2015). Determination of respiratory point compensation in healthy adults: can non-invasive near-infrared spectroscopy help?. J Sci Med Sport.

[48] Garrard CS, Emmons C (1986). The reproducibility of the respiratory responses to maximum exercise. Respiration.

[49] Barron A, Dhutia N, Mayet J, Hughes AD, Francis DP, Wensel R (2014). Test-retest repeatability of cardiopulmonary exercise test variables in patients with cardiac or respiratory disease. Eur J Prev Cardiol.

[50] Myers J, Goldsmith RL, Keteyian SJ, Brawner CA, Brazil DA, Aldred H (2010). The ventilatory anaerobic threshold in heart failure: a multicenter evaluation of reliability. J Card Fail.

[51] Garrard CS, Das R (1987). Sources of error and variability in the determination of anaerobic threshold in healthy humans. Respiration.

[52] Kothmann E, Danjoux G, Owen SJ, Parry A, Turley AJ, Batterham AM (2009). Reliability of the anaerobic threshold in cardiopulmonary exercise testing of patients with abdominal aortic aneurysms. Anaesthesia.

[53] Simmelink EK, Wempe JB, Geertzen JH, Dekker R (2009). Repeatability and validity of the combined arm-leg (Cruiser) ergometer. Int J Rehabil Res.

[54] Billinger SA, Loudon JK, Gajewski BJ (2008). Validity of a total body recumbent stepper exercise test to assess cardiorespiratory fitness. J Strength Cond Res..

[55] Billinger SA, VAN Swearingen E, McClain M, Lentz AA, Good MB (2012). Recumbent stepper submaximal exercise test to predict peak oxygen uptake. Med Sci Sports Exerc..

[56] Bulthuis Y, Drossaers-Bakker W, Oosterveld F, van der Palen J, van de Laar M (2010). Arm crank ergometer is reliable and valid for measuring aerobic capacity during submaximal exercise. J Strength Cond Res..

[57] Saitoh M, Matsunaga A, Kamiya K, Ogura MN, Sakamoto J, Yonezawa R (2005). Comparison of cardiovascular responses between upright and recumbent cycle ergometers in healthy young volunteers performing low-intensity exercise: assessment of reliability of the oxygen uptake calculated by using the ACSM metabolic equation. Arch Phys Med Rehabil.

[58] Davis JA, Sorrentino KM, Ninness EM, Pham PH, Dorado S, Costello KB (2006). Test-retest reliability for two indices of ventilatory efficiency measured during cardiopulmonary exercise testing in healthy men and women. Clin Physiol Funct Imaging.

